# Development and validation of a formula for objective assessment of cervical vertebral bone age

**DOI:** 10.1186/s40510-020-00338-0

**Published:** 2020-10-12

**Authors:** Raghavan Chandrasekar, Shyamala Chandrasekhar, K. K. Shantha Sundari, Poornima Ravi

**Affiliations:** 1 Chandroo Dental Clinic, no.40, K.B Dasan road, Teynampet, Chennai, 600018 India; 2 Department of Orthodontics and Dentofacial Orthopedics, Saveetha Dental College, Saveetha Institute of Medical and Technical Sciences, Saveetha University, Chennai, 600077 India; 3 Department of Oral and Maxillofacial Surgery, Saveetha Dental College, Saveetha Institute of Medical and Technical Sciences, Saveetha University, Chennai, India

**Keywords:** Bone age measurement, Cervical vertebral bone age, Skeletal age, Lateral cephalogram, Cervical vertebrae, Hand-wrist bones, Tanner-Whitehouse method

## Abstract

**Background and aim:**

Determination of skeletal maturity and bone age from cervical vertebrae has been well documented. Most methods described use subjective evaluation of morphological characteristics of cervical vertebrae and may be prone to variability and error. A few objective methods have also been developed, specific for certain populations and genders. The aim of this study was to establish and validate an objective method to determine cervical vertebral bone age from lateral cephalometric radiographs, for Asian South Indian patients of both genders.

**Methods:**

Ninety boys and 90 girls between 9 and 15 years of age were recruited, and their lateral cephalograms were taken. Using measurements made from the third and fourth cervical vertebrae, a formula to determine cervical vertebral bone age was derived using stepwise regression analysis. To validate the formula, a separate sample of 30 boys and 30 girls was chosen, and hand-wrist radiographs and lateral cephalograms were obtained. Cervical vertebral bone age (CVBA) was determined by applying the formula derived. Bone age was also calculated using the Tanner-Whitehouse 3 method. The bone ages determined by both methods were compared to each other and chronological age, using one-way ANOVA, Tukey’s post hoc analysis, and Pearson’s correlation coefficient.

**Results:**

The formulae derived in the current study to determine CVBA differed for both genders. No statistically significant difference was found between CVBA, bone age derived by the Tanner-Whitehouse 3 method, and chronological age for both boys (*p* value = 0.425) and girls (*p* value = 0.995). A moderate to strong positive correlation was found between CVBA, bone age, and chronological age.

**Conclusion:**

The formulae derived in this study were validated and are reliable for objectively determining cervical vertebral bone age and skeletal maturation from lateral cephalograms for Asian South Indian patients of both genders.

## Background

Craniofacial growth is an integral part of orthodontic diagnosis and treatment planning. Growth is characterized by variation in the amount, rate, time, pattern, and progress towards maturity [1]. Evaluation of individual growth status, and predicting periods of accelerated growth, such as the pubertal growth spurt, is essential for treatment planning and can influence treatment outcomes in growth modulation procedures and dentofacial orthopedics [2]. For instance, correctly timing functional appliance treatment during the patient’s accelerated growth period would provide the most optimal results in the correction of skeletal discrepancies [3].

The developmental status of a growing child can be assessed by various indicators, including chronological age, dental development, secondary sexual characteristics, peak height velocity, and skeletal maturation [2–5]. Chronological age is unreliable for assessment of developmental status because of the wide variation in timing and duration of the pubertal growth spurt and other developmental stages [6]. Radiographic assessment of the hand-wrist bones, by evaluation of ossification stages, is a reliable indicator of skeletal maturation and is found to be closely related to growth spurt [7–10]. Its main drawback, however, is that an additional radiograph is required [6, 11]. Hand-wrist radiographs cannot be taken in newer imaging systems such as the EOS scanner, which has the advantage of minimizing radiation dose [12].

To reduce both radiation exposure and diagnostic cost to the patient, assessment of cervical vertebral maturation, as seen in routine lateral cephalograms, has been explored. Lamparski [13] was the first to suggest that morphological changes occurring in cervical vertebral bodies during growth could be used to assess skeletal maturation. He found that this method was a reliable and valid alternative to radiographic assessment of hand-wrist bones for determination of skeletal age, and this has been substantiated by several authors [3, 14–17].

Since then, cervical vertebral maturation method has been increasingly used to determine skeletal maturation in dentofacial orthopedics, without the need for hand-wrist radiographs [2, 3, 18]. However, these studies were based on subjective evaluation, where cervical vertebrae were evaluated comparing the patients’ radiographic images with a standard atlas [6, 13]. There are concerns that these methods may be prone to interoperator variability and error [6]. Objective methods of evaluation have been developed by certain authors using regression formulae based on ratios of measurements in the third and fourth cervical vertebral bodies [19, 20]. However, these formulae have been shown to vary with gender and racial origin [6].

Till date, there is inadequate literature on objective evaluation of CVBA in the Asian South Indian population. The aim of the present study, therefore, was to establish and validate a formula in this population group that would determine the skeletal age from cervical vertebral maturation indicators.

## Materials and methods

The present prospective cohort study was designed to derive a formula to determine cervical vertebral bone age for boys and girls of South Indian origin and validate the same. Ethical committee clearance was granted by the Institutional Review Board of Saveetha University. The sample for the study was chosen from patients visiting the Department of Orthodontics, Saveetha Dental College, for orthodontic treatment. Patients in the age group of 9 to 15 years, in good general health and of South Indian origin, were included for the study. Patients who had a history of trauma, surgical intervention, or severe systemic illness or those who showed malformation of the cervical vertebrae or hand bones were excluded. Eligible patients were recruited serially until the desired sample size was achieved. The nature of the study was explained to the patients and their parents, and informed consent was obtained. As this study was restricted to Asian South Indians, patients who had one or both parents belonging to other ethnicities were also excluded.

The study consisted of two phases. In the first phase, we attempted to derive a formula for determining the cervical vertebral bone age for both boys and girls. For this part, a total of 180 patients (90 boys and 90 girls), in the age group of 9–15 years were recruited. Patients were divided into six age groups (9–10, 10–11, 11–12, 12–13, 13–14, 14–15 years) with 30 patients in each group (15 boys and 15 girls). For all patients, digital lateral cephalograms were taken (Planmeca Promax Digital radiographic machine, AGFA Drystar 5300 printer).

**Fig. 1:**
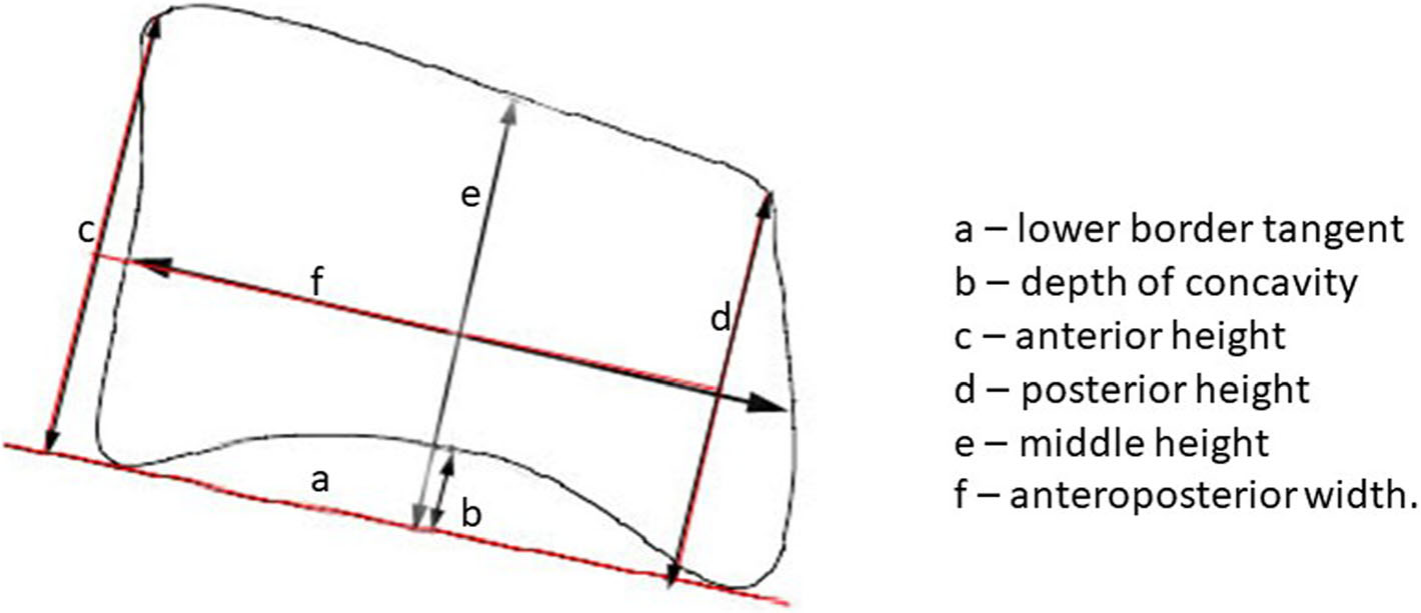
Measurements made on the C3 and C4 cervical vertebrae

Cephalometric analysis was done according to the method described by Mito et al. [19]. The outlines of C3 and C4 were traced using a 3H lead pencil on 0.03″ matte acetate paper, and all measurements were made using micrometer calipers. The following parameters were measured for both the vertebrae: anterior, middle, and posterior vertebral body heights and the anteroposterior body length. The depth of concavity was also assessed, as the distance between the deepest point of the curvature on the lower border to the lower border tangent (Fig. 1). All measurements were done by the same operator and were repeated after 10 days to assess for reliability. Intra-operator error between the two measurements was assessed using Dahlberg’s formula.

Using the above measurements, seven ratios were calculated (Table 1). Pearson’s correlation test was applied to these ratios and the patient’s chronological age. The ratios which showed higher correlation with chronological age were used to derive a formula for objectively determining cervical vertebral bone age. This formula was then derived separately for boys and girls, through stepwise multiple regression analysis, using the selected ratios as independent variables and mean chronological age as the dependent variable.

**Table 1:** Seven ratios that were assessed from the cephalogram

Ratio	Parameters assessed
AH/AP	Anterior height/anteroposterior width
PH/AP	Posterior height/anteroposterior width
H/AP	Middle height/anteroposterior width
AH/PH	Anterior height/posterior height
AH/H	Anterior height/middle height
H/PH	Middle height/posterior height
CONC/H	Depth of concavity/middle height

The ratios were named according to the cervical vertebra measured; for instance, AH3/PH3 refers to third cervical vertebra, while AH4/PH4 refers to the fourth vertebra

The second phase of the study attempted to validate the formula that was derived in the first phase of the study. A different sample of sixty patients of both genders, in the age group of 9–15 years, was chosen from patients visiting the Department of Orthodontics, Saveetha Dental College. The inclusion criteria were the same as that of part 1 of the study. The sample was subdivided into two groups, 30 each for boys and girls. Digital lateral cephalograms and hand-wrist radiographs (Wipro GE CARES DX525 radiographic machine) were taken for all 60 patients.

All the 60 lateral cephalograms were analyzed. Cervical vertebral bone age (CVBA) was determined by applying the formulae derived during the first part of the study for both boys and girls. Hand-wrist radiographs were evaluated to determine bone age by Tanner and Whitehouse method (TW3). Specific ossification centers of the radius, ulna, selected metacarpals, and phalanges were assessed, leading to their classifications into one of several stages. The scores were derived from each bone stage and calculated to compute the skeletal age or bone age.

The one-way ANOVA test was applied to identify differences between both the methods (cervical vertebral bone age and hand-wrist bone age) and the chronological age. To identify specific differences, the Tukey’s post hoc test was used. The relationship between the cervical vertebral bone age and the other two groups was also determined using Pearson’s correlation coefficient.

All the above statistical analysis was done using SPSS version 15. The study power was set at 80% and a *p* value of less than 0.05 was considered as significant (*α* error—95%).

## Results

**Fig. 2:**
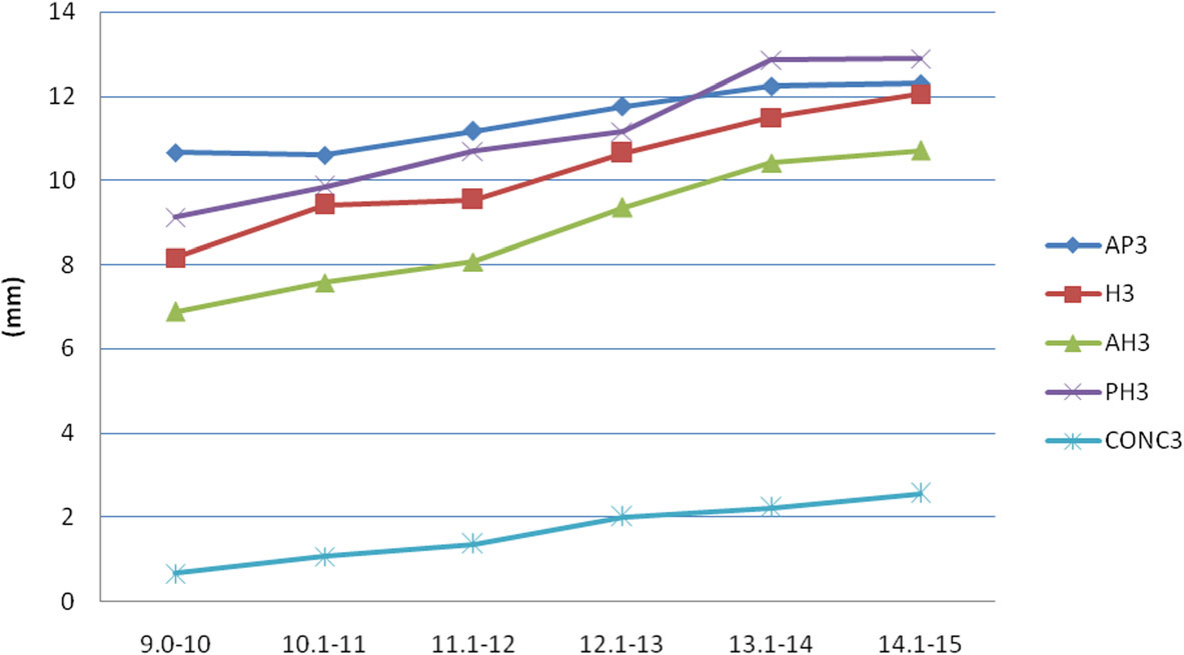
Changes in the parameters of C3 in females (AH, anterior height; PH, posterior height; H, middle height; AP, anteroposterior length; CONC, depth of concavity)

**Fig. 3:**
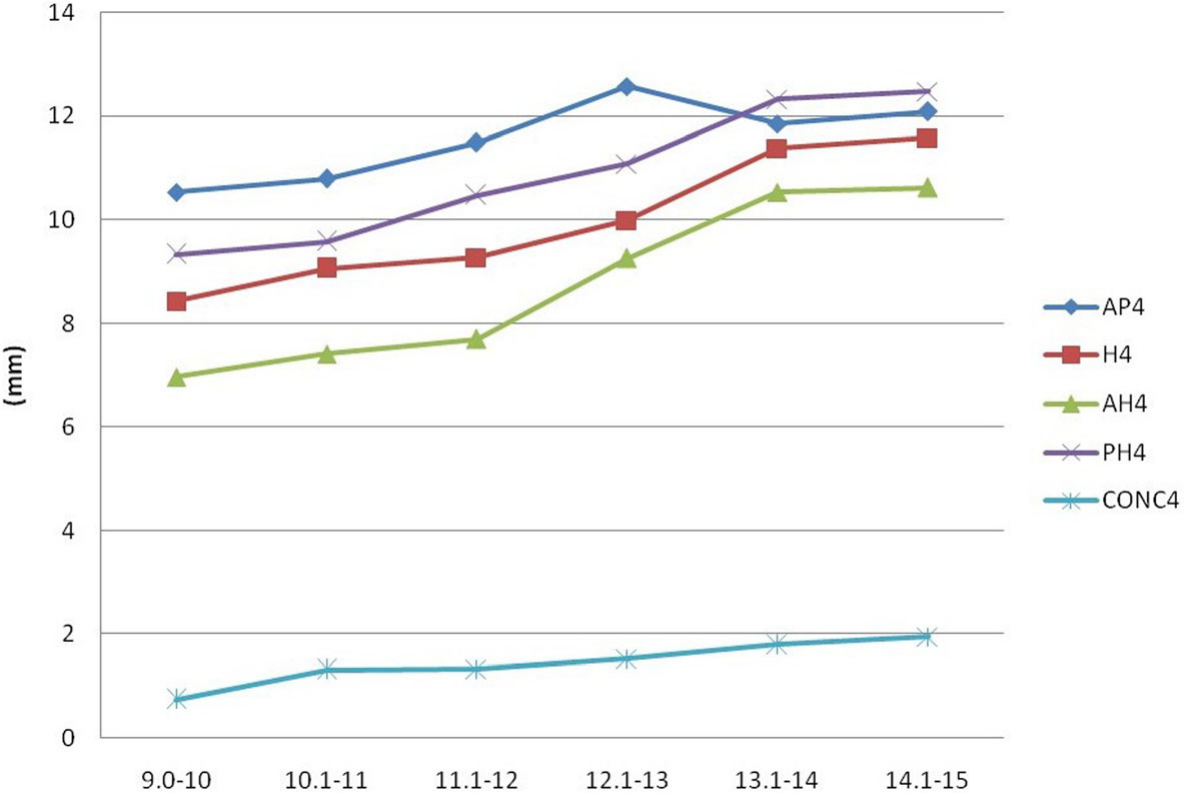
Changes in the parameters of C4 in females (AH, anterior height, PH, posterior height; H, middle height; AP, anteroposterior length; CONC, depth of concavity)

**Fig. 4:**
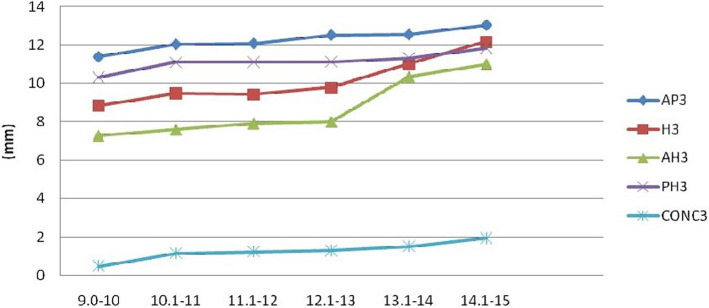
Changes in the parameters of C3 in males (AH, anterior height; PH, posterior height; H, middle height; AP, anteroposterior length; CONC, depth of concavity)

**Fig. 5:**
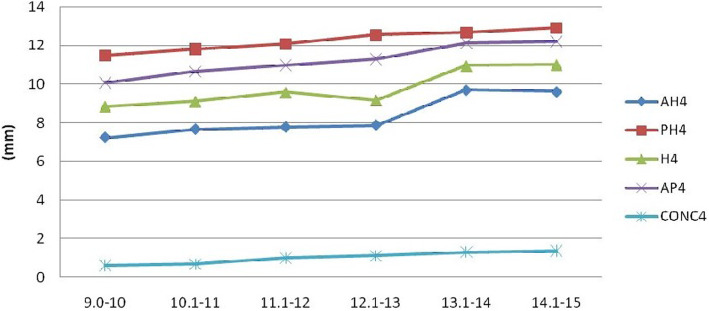
Changes in the parameters of C4 in males (AH, anterior height; PH, posterior height; H, middle height; AP, anteroposterior length; CONC, depth of concavity)

Analysis of the various parameters measured on third and fourth cervical vertebrae (Figs. 2, 3, 4, and 5) revealed that the depth of the concavity was very minimal between 9 and 10 years of age in both C3 and C4 among both boys and girls. The concavity gradually increased reaching a maximum between 13 and 14 years of age with minimum change till 15 years of age. This trend in growth was repeated with most of the other parameters, including anterior, middle, and posterior vertebral height and anteroposterior length of both C3 and C4 vertebrae. However, it was noted that the accelerated growth was distinct and pronounced in the posterior height of C3 in girls between 13 and 14 years of age, while for boys it was pronounced in the anterior and middle vertebral height.

Dahlberg’s analysis for intra-operator reliability gave values between 0.092 and 0.599. These values indicated adequate accuracy of measurement and reliability (DV < 1).

### Deriving a formula to calculate cervical vertebral bone age

Among the seven ratios established between the parameters measured for boys and girls separately, only five ratios in both genders showed a higher level of correlation with chronological age and were included in the formulae for determination of cervical vertebral bone age (CVBA). A gender variation was evident in the ratios selected for multiple regression analysis. Therefore, two separate formulae were derived to determine cervical vertebral bone age for boys and girls, through stepwise multiple regression analysis.

Cervical vertebral bone age for girls: 0.774 + (4.033 × PH_3_/AP_3_) − (0.087 × H_3_/AP_3_) + (2.26 × AH_3_/AP_3_) − (2.126 × AH_4_/AP_4_) + (8.513 × AH_4_/H_4_)

Cervical vertebral bone age for boys: 7.137 + (3.695 × AH_3_/AP_3_) − (1.582 × H_3_/AP_3_) + (8.716 × CONC_3_/H_3_) + (1.753 × AH_4_/AP_4_) + (1.604 × H_4_/AP_4_)

### Validation of the formula

The results of comparisons between the cervical vertebral bone age, hand-wrist bone age, and chronological age are illustrated in Tables 2 and 3. The one-way ANOVA test did not show any significant difference between the three groups. When specific differences between each group were compared, Tukey’s post hoc analysis also could not demonstrate any significant difference.

**Table 2:** One-way ANOVA test comparing the calculated cervical vertebral bone age, hand-wrist bone age, and chronological age

	Females			Males		
	Cervical vertebral bone age	Hand-wrist bone age	Chronological age	Vertebral bone age	Hand-wrist bone age	Chronological age
No. of patients	30	30	30	30	30	30
Range	9.35–13.16	9.8–13.5	9–15	9.36–12.76	9.1–14.7	9–15
Mean (years)	11.2785	11.2566	11.3006	11.2013	11.16	11.8136
Standard deviation	0.8024	2.2644	1.6029	2.2026	2.4975	2.2275
*p* value	0.995			0.425

**Table 3:** Tukey’s ad hoc analysis comparing cervical vertebral bone age, hand-wrist bone age, and chronological age

Comparison between			Significance (females)	Significance (males)
Cervical vertebral bone age	vs	Hand-wrist bone age	0.999	0.399
		Chronological age	0.999	0.879
Hand-wrist bone age	vs	Cervical vertebral bone age	0.999	0.399
		Chronological age	0.994	0.694
Chronological age	vs	Cervical vertebral bone age	0.999	0.879
		Hand-wrist bone age	0.994	0.694

The correlation between cervical vertebral bone age and the other two groups is shown in Table 4. The values indicated that there was moderate to strong correlation between the cervical vertebral bone age and hand-wrist bone age, as well as the chronological age.

**Table 4:** Extent of correlation between different ages

Groups	Correlation coefficient (females)	Correlation coefficient (males)
Cervical vertebral bone age and hand-wrist bone age	0.408	0.674
Cervical vertebral bone age and chronological age	0.505	0.598

## Discussion

The current study derived a formula to objectively determine cervical vertebral bone age (CVBA) in the Asian South Indian population. Similar objective methods of determining CVBA have been utilized by previous researchers, in other ethnic groups. Mito et al. [19] was the first to suggest an objective method for determining CVBA, and their formula was derived for Japanese girls. However, when this formula was applied to Brazilian patients, Caldas et al. [6] noted that it was reliable only for Brazilian girls. Subsequently, they developed different formulae for both genders and validated these for use in the Brazilian population [20]. Kumar et al. also attempted to use Mito’s formula for determining CVBA in the Asian North Indian population [21] and observed that it was reliable only for female patients. They stressed the need for developing a separate formula for males. Varshosaz et al. [22] derived a slightly different formula for the Iranian population.

The slight differences in formulae for the above studies could be explained by the fact that growth patterns tend to vary with race and gender. Zhang et al. [23] showed that ethnic and racial differences can affect growth patterns, and with subjective methods, bone age was overestimated in Asian and Hispanic populations. Therefore, it becomes necessary to identify objective methods of evaluating bone age specific to each ethnic group. Till date, no study exists for objectively evaluating cervical vertebral bone age in the Asian South Indian population. Indians, particularly South Indians, constitute one of the world’s most significant diaspora. Deriving a specific formula for this population would be very relevant in the global scenario.

Although skeletal maturation usually occurs in all seven vertebrae, only the vertebral bodies of C3 and C4 were selected for measurements. The first cervical vertebra is not clearly visible, and the second cervical vertebra shows minimal morphological changes. Cervical vertebrae below C4 cannot be visualized when a thyroid protection collar is worn during radiation exposure [6, 19, 20]. To derive the formula, cervical vertebral ratios that were found to most closely correlate with the patient’s chronological age were used. For both phases of the study, we chose patients who were between 9 and 15 years of age, as this age range corresponds to prepubertal and pubertal growth phase in most patients, and this is when most patients seek orthodontic treatment. The same age range in both phases also ensured reliability of the formulae. We also analyzed male and female patients as separate groups. This was done to account for the differences in the timings of morphological changes in the cervical vertebrae between genders.

In the current study, for both genders, growth acceleration occurred between 13 and 14 years of age. This was reflected through changes in the anterior, middle, and posterior heights, as well as antero-posterior lengths. A distinct and pronounced growth was also noted in the posterior height of C3 in girls and anterior and middle height of C3 in males between 13 and 14 years of age. These findings contrast with those of Caldas et al., who noted that in their study, accelerated growth occurred in anterior, middle, and posterior height of C3 and C4 from 10 to 13 years in females [20]. In males, growth occurred in these regions only in C3, from 12 to 15 years of age, while there was no change in C4. Mito et al., on the other hand, observed that accelerated growth in the anterior, middle, and posterior height occurred between 10 and 13 years of age, in both C3 and C4 of girls [19]. These findings reflect that there is clear ethnic and gender variation in growth of the C3 and C4 cervical vertebrae.

The formula derived by stepwise regression analysis was found to be substantially different from other studies. For Japanese girls, the formula included ratio of anterior height to anteroposterior length and anterior height to posterior height [19]. The study in the Brazilian population focused on ratios of anterior height and middle height to anteroposterior length [20]. In the Iranian population, anterior vertical height alone was found to be a strong predictive factor. In the current formula, however, apart from these parameters, the ratio of the posterior height to anteroposterior length and the ratio of the lower border concavity to the middle height were also taken into account. The depth of concavity was not measured by the previous authors [19, 20, 22]. However, Roman et al. reported that lower border concavity of cervical vertebrae was the best morphological vertebral parameter to estimate skeletal maturation [11]. Therefore, it was measured in the current study, and its ratio with middle vertebral height yielded a significant correlation.

For validating the formula, the Tanner-Whitehouse 3 method was used for evaluation of bone age from hand-wrist radiographs [24]. The Tanner-Whitehouse 3 method is reproducible and reliable and is not as dependent on subjective evaluation as the Greulich and Pyle method, which involves comparison with an atlas [9]. It also allowed for easy comparison of estimated bone age with bone age calculated from cervical vertebrae. The current study showed good correlation between the values derived from both methods. The formulae established in the present study are therefore reliable for objectively determining cervical vertebral bone age and skeletal maturation from lateral cephalograms of Asian South Indian patients of both genders.

Establishing skeletal bone age from cervical vertebrae can in turn predict other variables that may be useful for treatment planning. For instance, Sato et al. found that mandibular growth potential could be accurately assessed based on CVBA, which was useful in planning the timing of treatment, and treatment options in patients with class 3 malocclusions [25]. Studies have also shown that CVBA is correlated with dental eruption [26] and dental maturation of the lower permanent canine and second molar [27]. This can aid in orthodontic treatment planning.

### Limitations and future directions

The main limitation of this study is that it was cross-sectional, and measurements for all patients were only taken at one point in time. A longitudinal study would have allowed further validation of the formula at different stages of maturation. However, this would have led to further radiation exposure and may have been prone to attrition bias.

Most formulae generated for calculating cervical vertebral bone age in different ethnic groups are cumbersome and prone to error if worked out manually. The next logical step, as suggested by Caldas et al. [21], would be to develop a software that could automatically calculate CVBA for different ethnic groups based on measurements obtained from cervical vertebrae on lateral cephalograms. Research has already begun in this direction. Kok et al. recently compared the accuracy of different artificial intelligence algorithms for assessing cervical vertebral maturation [28]. However, a common platform that would include formulae for different ethnicities is needed; the world today is a global village and it is not uncommon for orthodontists to encounter young patients from different ethnic groups and populations.

In conclusion, the present study highlights that skeletal maturation indicators have slight variations across diverse ethnic groups, and may require different methods of objective evaluation. The formula derived in the current study has been validated for the Asian South Indian population and may be effectively used to determine cervical vertebral bone age from lateral cephalograms in this population.

## Data Availability

The datasets used and/or analyzed during the current study are available from the corresponding author on reasonable request.

## Abbreviations

CVBA: Cervical vertebral bone age
TW3: Tanner-Whitehouse 3 method
C2, C3, C4: Second, third, and fourth cervical vertebrae
AH: Anterior height of cervical vertebral body
PH: Posterior height of cervical vertebral body
H: Middle height of cervical vertebral body
AP: Anteroposterior length of cervical vertebral body
CONC: Depth of concavity of cervical vertebral body
